# Infektion durch Ostseewasser erfordert großflächige Mesh-Haut-Transplantation am Universitätsklinikum Ulm

**DOI:** 10.1007/s00113-024-01501-6

**Published:** 2024-11-25

**Authors:** F. Kreß, P. Schenk, F. Gebhard, K. Schütze

**Affiliations:** Abteilung für Unfall- , Hand‑, Plastische und Wiederherstellungschirurgie, Universitätsklinikum Ulm, Albert-Einstein-Allee 23, 89070 Ulm, Deutschland

**Keywords:** *Vibrio vulnificus*, Ostsee, Nekrose, Septischer Schock, Mesh, *Vibrio vulnificus*, Baltic Sea, Necrosis, Septic shock, Mesh

## Abstract

*Vibrio vulnificus* (*V. vulnificus*) ist ein gramnegatives Bakterium, das schwere Infektionen verursachen kann, wenn es über offene Wunden oder den Verzehr von Schalentieren in den menschlichen Körper gelangt. Vermehrtes Auftreten der Bakterien ist bei Wassertemperaturen > 20 °C bekannt. Außerdem ist ein geringer Salzgehalt von 5–25 ‰ für das Vermehren von Vibrionen förderlich. In der Übersichtsarbeit von Fleischmann et al. wird der direkte Zusammenhang von niedrigem Salzgehalt und hoher Wassertemperatur mit dem vermehrten Auftreten von *V. vulnificus* an deutschen Ostseeküsten aufgezeigt [7]. Es kann zu einer fulminanten nekrotisierenden Fasziitis kommen, die einen septischen Schock, eine Amputation und den Tod zur Folge haben kann.

Dieser Fallbericht zeigt die erfolgreiche chirurgische Behandlung einer fulminanten Unterschenkelinfektion mit septischem Schock nach Kontakt mit Ostseewasser in Deutschland. Nach einem schnellen und großflächigen Débridement folgte die erfolgreiche Transplantation von Hautgewebe mittels Mesh-Graft.

## Abstract (en)

*Vibrio vulnificus* (*V. vulnificus*) is a gram-negative bacterium that can cause severe infections after entering the human body via open wounds or consumption of shellfish. An increased occurrence of bacteria is known at water temperatures > 20 °C. In addition, a low salt content of 5–25‰ is beneficial for the reproduction of *Vibrio* spp. In the review by Fleischmann et. al. the direct correlation between low salinity and high water temperature with the increased occurrence of *V. vulnificus* on German Baltic Sea coasts was demonstrated [7]. It can cause fulminant necrotizing fasciitis which can result in amputation, septic shock and death. This case report shows the successful surgical treatment in Germany of a fulminant lower leg infection with septic shock after coming into contact with Baltic Sea water. A rapid and extensive debridement was followed by the successful transplantation of cutaneous tissue using a mesh graft.

## Fallbericht

### Anamnese und Befund

Eine 66-jährige Patientin stellte sich in der Notaufnahme eines Grund- und Regelversorgers mit Schmerzen, Rötung und plötzlich zunehmender Schwellung ihres rechten Unterschenkels vor. Ein direktes Trauma sowie lange Flugreisen oder ein Auslandsaufenthalt wurden verneint.

Bei erhöhten laborchemischen Infektparametern und dem klinischen Bild eines nekrotisierendem Weichteilinfektes (NSTI) erfolgten eine Abszessspaltung sowie Einlage eines Vakuumversiegelungssystems (VVS). Zudem wurde bei knöcherner konsolidierter distaler Fibulafraktur das einliegende Osteosynthesematerial entfernt.

Postoperativ zeigt die Patientin trotz chirurgischer Therapie und empirischer antibiotischer Therapie (Clindamycin 600 mg 3‑mal tgl. i.v.) klinische Anzeichen eines septischen Schocks mit akutem Nierenversagen, woraufhin die Patientin zur weiteren Therapie und Stabilisierung des Allgemeinzustandes ans Universitätsklinikum verlegt wurde (CRP 282 mg/l (< 5 mg/l), Leukozytenzahl 15,4 (4,4–11,3 Giga/l) PCT > 100.000 *µ*g/l (< 0,046 *µ*g/l), Kreatinin 335 *µ*ml/l (45–84 *µ*ml/l)).

### Therapie und Verlauf

Im Rahmen der Erstvorstellung im Haus der Grund- und Regelversorgung erfolgte bei Unterschenkelabszess zunächst die Abszessspaltung, Entfernung des Osteosynthesematerials sowie ein chirurgisches Débridement medial und lateral am Unterschenkel mit Anlagen einer Vakuumversiegelung (VVS). Intraoperativ zeigte sich das klinische Bild einer nekrotisierenden Fasziitis (NSTI). Postoperativ zeigte sich die Patientin im septischen Schock und wurde bei zusätzlicher Verschlechterung des Lokalbefundes notfallmäßig in die chirurgische Intensivstation des Universitätsklinikums verlegt. Bei klinischer Verschlechterung wurde die antibiotische Therapie mit Piperacillin/Tazobactam erweitert.

Nach Verlegung zeigt die Patientin eine kontinuierliche Verschlechterung der Hämodynamik und Nierenfunktion. Zur Behandlung des septischen Schocks wurde eine Therapie mit Katecholaminen und Hydrocortison eingeleitet.

Das mikrobiologische Ergebnis der ersten chirurgischen Probenahme zeigt das Vorhandensein von *V. vulnificus* (Infobox 1). In der bereits im externen Krankenhaus abgenommen Blutkultur zeigte sich ebenfalls der Nachweis von *V. vulnificus*.

Nachdem der Erreger der nekrotisierenden Fasziitis und Sepsis detektiert wurde, eskalierten wir die Antibiotikatherapie durch die Zugabe von Doxycyclin. Piperacillin/Tazobactam konnte nach 6 Tagen abgesetzt werden (Fortführung der Therapie mit Clindamycin und Doxycyclin).

Bei der ausführlicheren Anamnese gab die Patientin an, vor einigen Tagen einen Badeurlaub an der Ostsee verbracht zu haben.

#### Infobox *V. vulnificus*

- *V. vulnificus* ist ein gramnegatives Bakterium, das in warmen Meeresgewässern mit niedrigem Salzgehalt wie Brackwasser oder in Küstennähe vorkommt.
- Für den Menschen existieren zwei Aufnahmewege: orale Aufnahme durch Nahrung oder Aufnahme über offene Wunden.
- Risikofaktoren für einen schweren Verlauf: männliches Geschlecht (90 %), ältere Personen > 40 Jahre (85 %), Patienten mit Lebererkrankungen, Diabetes und Immuninsuffizienz [1].
- Verzögertes chirurgisches Débridement erhöht die Mortalität. Todesfälle in Europa wurden berichtet [2, 3].
- Trotz raschem chirurgischem Débridement und antibiotischer Therapie ist ein Erhalt der betroffenen Extremitäten nicht gesichert [4].
- Die antibiotischte Therapie sollte mit Cephalosporinen und 3.-Generation-Tetrazyklinen erfolgen.
- Der Klimawandel, mit Erwärmung der See und Abnahme des Salzgehaltes, begünstigt das Auftreten von *V. vulnificus* auch an deutschen Küstengebieten [5, 6].

**Abb. 1:**
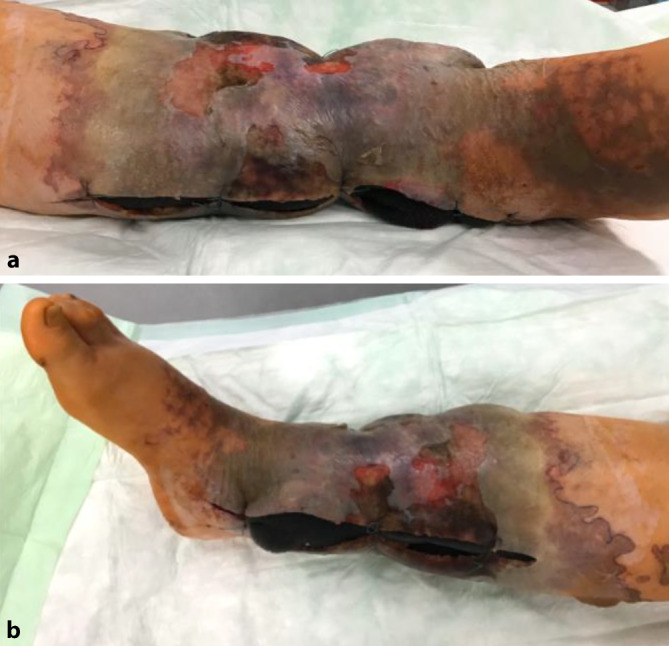
Präoperative Situation: rechte untere Extremität mit großflächigen Nekrosen. **a** Laterale Inzision, **b** mediale Inzision

Am Tag der Verlegung führten wir ein erneutes radikales chirurgisches Débridement mit Nekrosektomie und Wechsel des VVS durch. Der Situs zeigt 2 etwa 40 cm lange Inzisionen am Innen- und am Außenknöchel von distal nach proximal. Die Haut zeigte bereits graue livide Verfärbungen mit Nekrosen und Blasen (Abb. 1). Das postoperative Labor zeigte weiterhin stark erhöhte Infektparameter bei fallender Tendenz: CRP 185 mg/l (< 5 mg/l), Leukozytenzahl 12,0 (4,4–11,3 Giga/l) PCT 23.900 *µ*g/l (< 0,046 *µ*g/l).

Die intraoperative mikrobiologische Probennahme aus dem tiefen Subkutangewebe sowohl medial auf Höhe des Malleolus medialis als auch lateral im Bereich der distalen Fibula am Universitätsklinikum Ulm bestätigte erneut das Vorhandensein von *V. vulnificus*.

Die antibiotische Therapie wurde nun durch Absetzen von Clindamycin und Hinzunahme von Ciprofloxacin ergänzt.

Vor unserem zweiten Eingriff im Sinne einer Third-Look-Operation erfolgte eine CT-Diagnostik mit Gefäßdarstellung.

Es zeigten sich keine weiteren Abszesse sowie Hinweise auf eine Osteomyelitis.

**Abb. 2:**
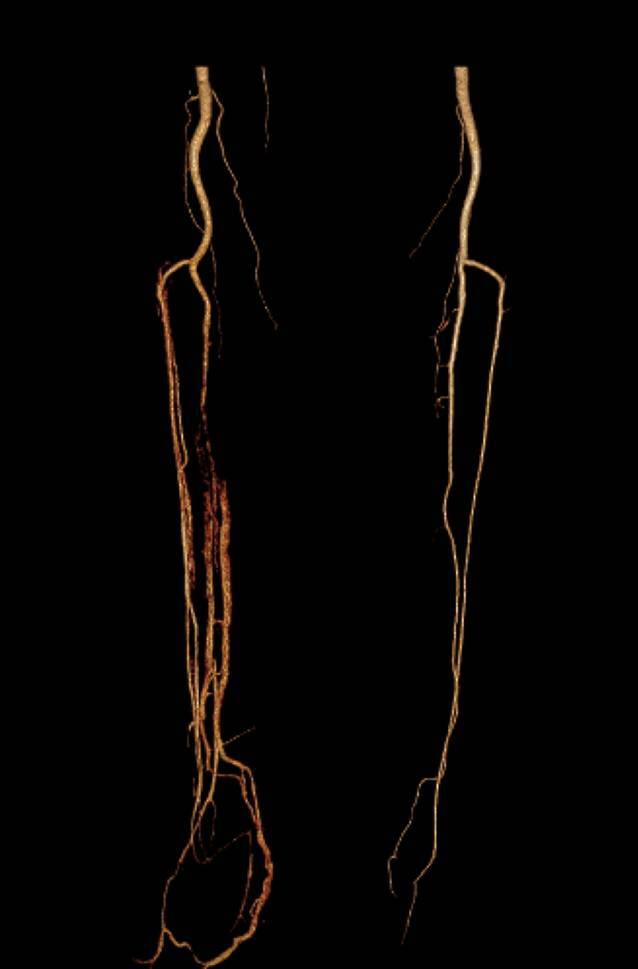
Beidseitige computertomographische Angiographie der unteren Extremitäten, a.-p.-Aufnahme

Die Gefäßversorgung zeigte sich durch eine Dreigefäßversorgung suffizient (Abb. 2).

In unserem Third-Look-Eingriff zeigte sich intraoperativ die Zunahme von nekrotisierendem Gewebe. Die Zugänge wurden nun konsequent erweitert.

Wir wiederholten das Débridement und den VVS-Wechsel an jedem 3. Tag.

**Abb. 3:**
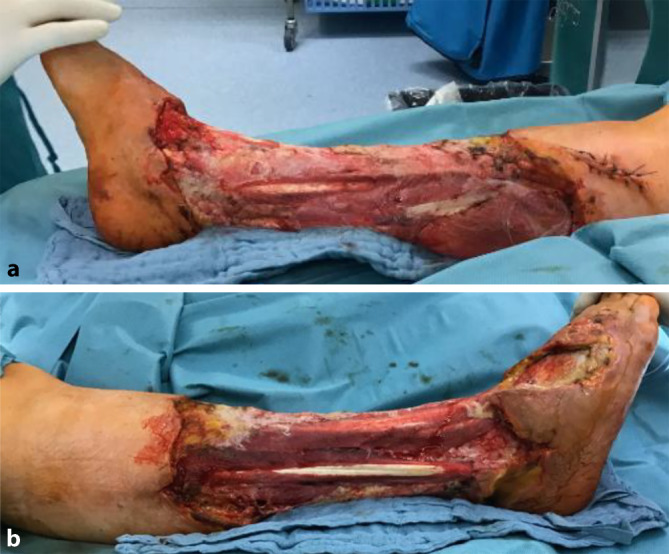
Postoperative Situation: **r**echte untere Extremität nach Nekrosektomie: **a** mediale Ansicht, **b** laterale Sicht

Wir besprachen mit der Patientin die Amputation der rechten unteren Extremität unterhalb des Knies bei mittlerweile nahezu freiliegendem Schienbein- und Wadenbeinknochen sowie der Mm. gastrocnemius, fibularis longus und tibialis anterior (Abb. 3).

Nach dem fünften Wunddébridement und VVS-Wechsel zeigten sich die Infektionsparameter fallend: CRP 69 mg/l (< 5 mg/l), Leukozytenzahl 10,0 (4,4–11,3 Giga/l) PCT 5010 *µ*g/l (< 0,046 *µ*g/l).

Das chirurgische Débridement zeigte sich erfolgreich, und in den mikrobiologischen Proben konnten keine Bakterien mehr nachgewiesen werden.

**Abb. 4:**
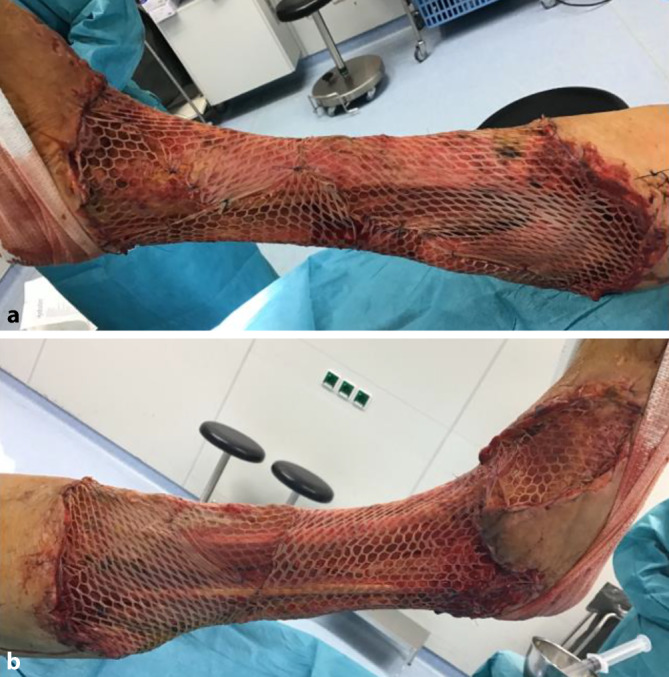
Postoperative Situation: **r**echte untere Extremität nach Mesh-Transplantation: **a** mediale Ansicht, **b** seitliche Ansicht

Zweieinhalb Wochen nach der stationären Aufnahme führten wir eine plastisch-chirurgische Behandlung mithilfe einer Spalthauttransplantation durch. Die Haut wurde vom lateralen Oberschenkel entnommen. Wir verwendeten ein Mesh-Verhältnis von 3:1. Laborchemisch zeigten sich nun nahezu normwertige Infektparameter und nur leicht erhöhte Retentionsparameter bei guter Miktion: CRP 29,6 mg/l (< 5 mg/l), Leukozytenzahl 7,0 (4,4–11,3 Giga/l) Kreatinin 158 *µ*ml/l (45–84 *µ*ml/l). Wir transplantierten das Netz zirkulär auf den Unterschenkel. Es war möglich, alle freiliegenden Gewebeteile zu decken (Abb. 4).

Fünf Tage nach der Mesh-Transplantation entfernten wir die Vakuumversiegelung auf dem Mesh-Graft. Es zeigte sich ein vollständig rosiges vitales Hauttransplantat. Bei Keimfreiheit konnte die antibiotische Therapie beendet werden (antibiotische Therapie nach Erregernachweis: 21 Tage Doxycyclin und Ciprofloxacin). Die Schmerzen waren erträglich. Die Nachsorge erfolgte durch regelmäßige sorgfältige Verbandwechsel und Pflege des Transplantats durch Auftragen einer Mischung aus Bepanthen und Jodcreme. Der Wundverbandwechsel und die Wundkontrolle erfolgten einmal täglich.

**Abb. 5:**
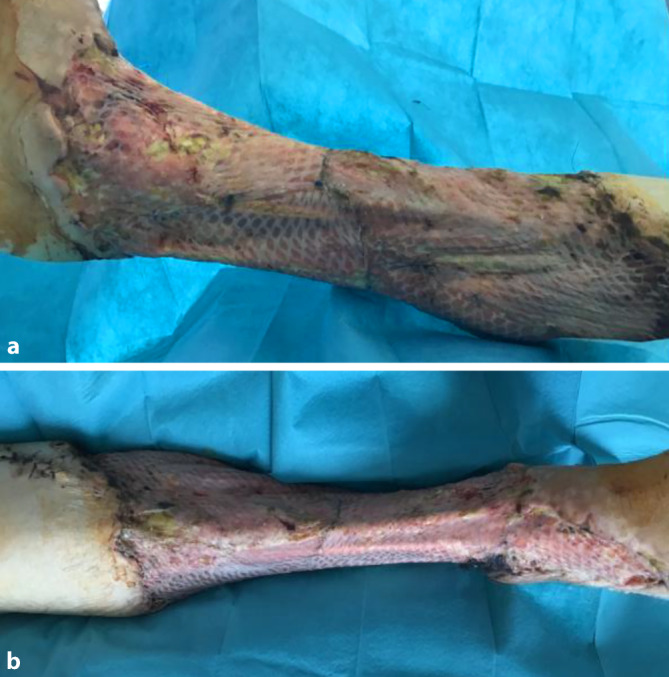
Rechte untere Extremität am 9. postoperativen Tag nach der Mesh-Transplantation: **a** Seitenansicht , **b** a.-p.-Ansicht

Abb. 5 zeigt das vitale Mesh-Transplantat am 9. postoperativen Tag.

Vier Wochen nach initialer Mesh-Transplantation wurden kleinere Areale am Außenknöchel am medialen proximalen Unterschenkel sowie der Peronäalsehne mittels Mesh-Graft erneut gedeckt.

Die neurologische Untersuchung vor der Entlassung zeigte lediglich eine leichte Kraftgradminderung der Fußheber und Fußstrecker 4/5. Sensible Defizite wurden in den oberflächigen sensiblen Hautnerven, N. peronaeus superficialis, N. suralis und R. calcaneus des N. tibialis beschrieben.

**Abb. 6:**
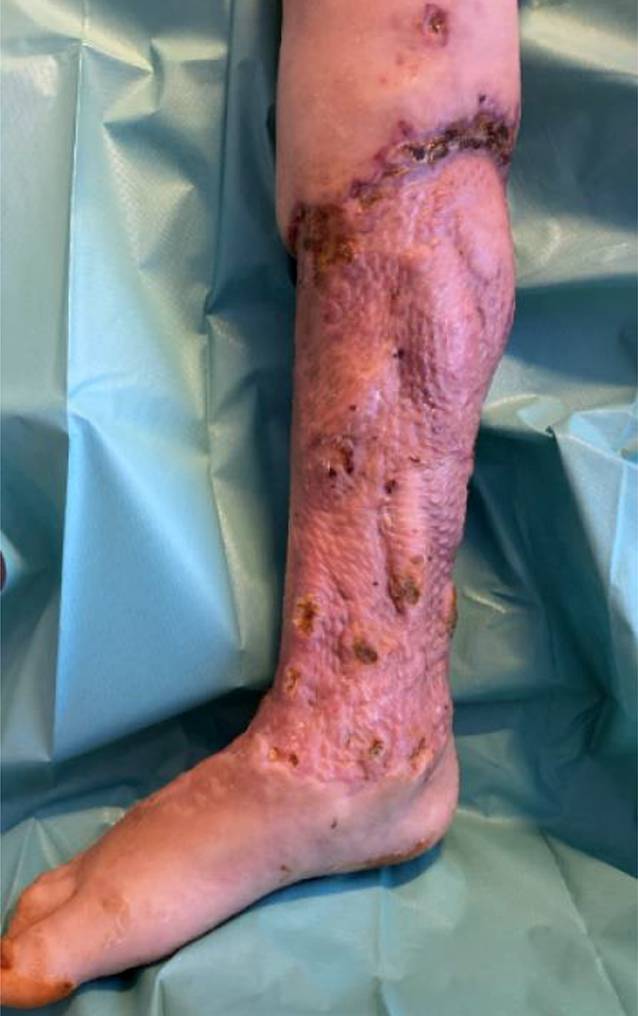
Vitales Mesh-Transplantat am Entlassungstag, mediale Ansicht der rechten unteren Extremität

Die Patientin konnte 55 Tage nach der Aufnahme mit schmerzfreiem flüssigem Gangbild an Unterarmgehstützen nach Hause entlassen werden. Das Mesh-Transplantat zeigte sich vital (Abb. 6).

## Diskussion

Dieser Fallbericht zeigt die erfolgreiche Behandlung einer fulminanten *V.-vulnificus*-Infektion mit fulminanter Nekrosenbildung der Haut und des Weichteilgewebes an der unteren Extremität bei einer gesunden 66-jährigen Frau in Deutschland nach Kontakt mit Wasser der Ostsee bei Wundheilungsstörung.

Durch ein rasches und gründliches chirurgisches Débridement in Kombination mit einer gezielten Antibiotikatherapie gelang es, das Leben der Patientin zu retten. Diese kritische Intervention erfolgte innerhalb der ersten 24 h nach Einsetzen des septischen Schocks und akuten Nierenversagens – ein Zeitfenster, in dem laut Fachliteratur bis zu 50 % der Betroffenen versterben [2].

Außerdem ist es gelungen, die Extremität der Patientin mit guter sensomotorischer Funktion zu erhalten.

Es wird auch in Zukunft wichtig sein, erhöhte Konzentrationen von potenziell humanpathogenen Bakterien und folglich Gesundheitsrisiken in marinen und küstennahen Gebieten effektiv zu überwachen. Hierzu wird u. a. die Wasserqualität an deutschen Küstengebieten regelmäßig überwacht. Hier gilt ein besonderes Augenmerk dem Salzgehalt und der Wassertemperatur.

In den Tropen treten Nicht-Cholera-Vibrionen ganzjährlich auf. An deutschen Küstengewässern zeigt sich hier eine starke Saisonalität. Überwiegend in den Sommermonaten, wenn die Wassertemperatur über 20 °C ansteigt, häuft sich die Anzahl der Vibrionen an deutschen Badestränden [5].

*Vibrio vulnificus* gedeiht in Wasser mit einem Salzgehalt von 0,5–2,5 % und bei Wassertemperaturen ab 18–20 °C. Die Überwachung dieser Umweltbedingungen ist entscheidend, da höhere Temperaturen und geeignete Salzgehalte die Vermehrung der Bakterien fördern [8]. Das European Centre for Disease Prevention and Control [9] veröffentlicht auf einer interaktiven Karte eine 5‑tägige Vorhersage über das mögliche Auftreten von Vibrionen [9, 10].

Seit 2020 besteht in Deutschland eine namentliche Meldepflicht für Infektionen durch humanpathogene Vibrionen nach dem Infektionsschutzgesetz (IfSG). Labore müssen Nachweise von Nicht-Cholera-Vibrionen gemäß § 7 Abs. 1 des IfSG melden, sofern der Nachweis auf eine akute Infektion hinweist.

Dieser Fall zeigt die zunehmende chirurgische Herausforderung der Therapie von Weichteilinfektionen, durch das bis dato seltene Auftreten von *V. vulnificus* an deutschen Ostsee- und Nordseestränden. Mit einer zunehmenden Inzidenz in den kommenden Jahren bei fortschreitender Klimaerwärmung ist zu rechnen.
